# Low-Latency Realism Through Randomized Distributed Function Computations: A Shannon Theoretic Approach †

**DOI:** 10.3390/e28010086

**Published:** 2026-01-11

**Authors:** Onur Günlü, Maciej Skorski, H. Vincent Poor

**Affiliations:** 1 Lehrstuhl für Nachrichtentechnik, Technische Universität Dortmund, 44227 Dortmund, Germany; 2 Information Theory and Security Laboratory (ITSL), Linköping University, 581 83 Linköping, Sweden; 3 Faculty of Information Technology, Czech Technical University in Prague, 160 00 Prague, Czech Republic; maciej.skorski@cvut.cz; 4 Department of Electrical and Computer Engineering, Princeton University, Princeton, NJ 08544, USA; poor@princeton.edu

**Keywords:** ultra-efficient semantic communications, randomized distributed function computation (RDFC), realism for generative modeling, realism for image compression, rate–distortion–perception (RDP)

## Abstract

Semantic communication frameworks aim to convey the underlying significance of data rather than reproducing it exactly, a perspective that enables substantial efficiency gains in settings constrained by latency or bandwidth. Motivated by this shift, we study the rate–distortion–perception (RDP) trade-off for image compression, a setting in which reconstructions must be not only accurate but also perceptually faithful. Our analysis is carried out through the lens of randomized distributed function computation (RDFC) framework, which provides a principled means of synthesizing randomness and shaping output distributions. Leveraging this framework, we establish finite-blocklength characterizations of the RDP region, quantifying how communication rate, distortion, and perceptual fidelity interact in non-asymptotic regimes. We further broaden this characterization by incorporating two practically relevant extensions: (i) scenarios in which encoder and decoder share side information, and (ii) settings that require strong secrecy guarantees against adversaries, which might include those with quantum capabilities. Moreover, we identify the corresponding asymptotic region under a perfect realism constraint and examine how side information, finite blocklength effects, and secrecy demands influence achievable performance. The resulting insights provide actionable guidance for the development of low-latency, secure, and realism-aware image compression and generative modeling systems.

## 1. Introduction

Semantic communication marks a shift in both the goals and the design principles of modern communication systems. Instead of enforcing a bit-accurate reconstruction of transmitted data, semantic approaches aim to convey the meaning of the information for the task at hand [1,2]. This paradigm becomes particularly beneficial in settings where latency or bandwidth is severely constrained, such as augmented/virtual reality, autonomous driving, and immersive media, because transmitting only the semantically essential components can drastically reduce the communication load while still supporting the intended functionality.

A natural way to formalize semantic communication is through the lens of remote source coding [3], ([4], p. 78), ([5], p. 118), where the receiver is required to compute a function of the source observed at the transmitter. Motivated by this viewpoint, we introduced the randomized distributed function computation (RDFC) framework in [6]. RDFC highlights the central role of controlled randomization in distributed computation: the encoder–decoder pair is designed so that the induced output distribution matches a desired target probability law. This viewpoint naturally accommodates applications such as neural compression based on generative models [7,8], federated learning with side information [9], and neural compression mechanisms that satisfy differential privacy constraints [6,10,11]. Rather than adding external noise, RDFC uses coding-theoretic strategies to shape the output distribution while preserving utility. This approach not only leads to major communication-rate reductions in latency-critical and privacy-sensitive environments, but also provides strong performance guarantees without requiring significant amounts of shared common randomness, since its guarantees hold on a per-instance basis [6,12,13].

The rate–distortion–perception (RDP) problem plays a central role in applications such as generative modeling [14] and image compression ([15], Section 17.4.2). Viewed through the lens of RDFC framework, RDP concerns the task of producing reconstructions that remain perceptually faithful to the original while respecting communication-rate limits [16,17,18,19,20,21]. In contrast to the standard rate–distortion formulation, the RDP problem incorporates an additional constraint that captures perceptual quality, reflecting the requirement that reconstructions align with human visual judgments. Practical systems often enforce this notion of realism using discriminator networks from generative modeling [22,23,24], which are trained to distinguish genuine samples from generated ones. For theoretical analysis, a widely adopted surrogate for perceptual fidelity is the requirement that the reconstruction distribution approximate the source distribution, as outlined in [25,26]. This distributional realism condition integrates naturally into the RDFC viewpoint: the coding mechanism synthesizes a decoder output that emulates a prescribed target probability law. Leveraging this connection, we use RDFC-based random coding methods to obtain finite-blocklength characterizations of the non-asymptotic RDP limits, with strong function computation guarantees that do not rely on large amounts of common randomness.

Here, we derive achievable non-asymptotic RDP bounds that describe the interplay among rate, distortion, and perceptual quality in the RDP setting. These finite-blocklength descriptions provide rigorous benchmarks for assessing the performance of practical compression schemes and offer insights relevant to systems operating under stringent latency requirements. We further broaden the analysis to include scenarios in which both terminals have access to side information (SI) correlated with the source. Such settings arise naturally in sequential or streaming applications, where earlier frames in an image sequence carry semantic information that is informative about the current input.

Distributed function computations become significantly more challenging when communication takes place over public channels, as unintended parties may gain access to sensitive information [27,28]. To mitigate such leakage in the RDP setting, we extend our coding-based constructions by incorporating techniques from physical-layer security (PLS). These methods restrict what an eavesdropper can infer from the transmitted information, strengthening confidentiality in neural compression systems where both the intended receiver and an adversary can observe the channel output. Unlike cryptographic mechanisms that rely on assumptions about computational hardness, PLS provides information-theoretic guarantees that remain robust even in the presence of quantum-capable adversaries [29,30]. Moreover, PLS has emerged as a key component of joint source–channel coding architectures for image transmission [31], making it a natural complement to the RDP models developed in this work.

In this context, we characterize secure RDP regions in both finite-blocklength and asymptotic regimes under a strong secrecy constraint, ensuring that the amount of leaked information remains negligible in a strong sense, rather than merely when normalized by blocklength that is common in weak secrecy formulations. Integrating RDFC-based coding tools with PLS thus creates a unified framework that simultaneously addresses perception, distortion, latency, and confidentiality. This synthesis bridges theoretical advances in distributed function computation with practical requirements in neural image compression, yielding communication strategies that preserve realism and efficiency while offering rigorous security guarantees. As a result, the proposed methodology aligns with the demands of emerging applications that require communication systems to be simultaneously low-latency, secure, and context-aware.

### 1.1. Main Contributions

The key contributions of this work are as follows.

- We establish non-asymptotic inner bounds for the RDP region to quantify the achievable rate requirements at finite blocklengths while ensuring high perceptual fidelity and low distortion;
- We extend these bounds to settings with information-leakage constraints, deriving achievable regions that guarantee strong secrecy without compromising distortion or perceptual performance;
- We identify the corresponding asymptotic secure RDP region under a perfect realism constraint, clarifying how this regime relates to its near-perfect counterpart.

Parts of the results listed above appeared in the conference version of this work in [16], and here we corrected a minor issue in the distortion expressions. Moreover, in this work, we additionally

- Establish the non-asymptotic RDP with SI regions, where shared SI is correlated with the encoder input;
- Consider a binary RDP example to illustrate the significant increase in the required amount of communication and randomness resources when a security constraint is imposed;
- Analyze the resulting rate regions, which includes (i) illustrating significant communication load reductions over classical data compression methods; (ii) identifying the main effects of secrecy constraints on RDP regions; (iii) illustrating significant communication and common-randomness rate gains from available SI; and (iv) highlighting the relationships between non-asymptotic and asymptotic results.

While prior work on the RDP trade-off has established asymptotic limits under various notions of realism, this paper focuses on regimes and resources that are left implicit in most existing analyses. In particular, we provide finite-blocklength achievable RDP regions and explicitly characterize the role of common randomness, noting that common randomness is in general necessary to satisfy stringent perceptual constraints in RDP formulations [32]. Our framework further enables systematic extensions to shared SI and strong secrecy constraints within the same RDP formulation.

### 1.2. Paper Organization

Section 2 introduces the point-to-point RDFC framework used throughout the paper and formalizes the non-asymptotic RDP regions considered, including the variants with SI and secrecy constraints. Section 3 then develops the main technical results, establishing three finite-blocklength achievable rate regions as well as a corresponding asymptotic secure RDP region. In Section 4, we compare these rate regions, their asymptotic limits, and classical data compression baselines. Finally, Section 5 discusses the overall insights gained from the analysis and outlines their broader scientific and technological implications.

### 1.3. Notation

Random variables are written in uppercase *X* and their realizations in lowercase *x*. The probability distribution of *X* is denoted by $P_{X}$ with support $\mathrm{supp}(P_{X})$. The calligraphic letters X denote sets, with |X| representing their cardinality. For a block of length *n*, we denote sequences as $X^{n}=\left(X_{1},X_{2},\ldots ,X_{i},\ldots ,X_{n}\right)$. If a sequence is independent and identically distributed (i.i.d.), then its joint distribution is $P_{X}^{n}\left(x^{n}\right)≜\prod_{i=1}^{n}P_{X}\left(x_{i}\right)$.

The total variation (TV) distance between two distributions $P_{Y}$ and $P_{X}$ over a common alphabet X is defined as

(1) $$\begin{array}{c} {∥P_{Y}-P_{X}∥}_{\mathrm{TV}}≜\frac{1}{2}\sum_{x\in X}\left|P_{Y}\left(x\right)-P_{X}\left(x\right)\right|. \end{array}$$

For a joint probability distribution $P_{XY}$, the information density is

(2) $$\begin{array}{c} ı\left(X,Y\right)=\log\frac{P_{XY}\left(x,y\right)}{P_{X}\left(x\right)P_{Y}\left(y\right)}. \end{array}$$

For distributions of the form $P_{XYZ}=P_{XZ}P_{Y}$, the corresponding information density is

(3) $$\begin{array}{c} ı\left(XZ,Y\right)=\log\frac{P_{XZY}\left(x,z,y\right)}{P_{XZ}\left(x,z\right)P_{Y}\left(y\right)}. \end{array}$$

We denote the Big O notation as O(·), and use Var[·] to denote variance and $Q^{-1}\left(\cdot \right)$ to denote the inverse *Q*-function, i.e., inverse of the tail distribution of a standard Gaussian. For any ℓ∈R, we have ${\left[\ell \right]}^{+}≜\max\left\{\ell ,0\right\}$. All logarithms are base 2. The interval [a:b] denotes the set of integers {a,a+1,…,b}, and ${\left\{\cdot \right\}}^{c}$ denotes a complementary event.

A sequence $x^{n}$ is δ-letter typical with respect to $P_{X}$, i.e., $x^{n}\in T_{\delta }^{n}\left(P_{X}\right)$, if we have

(4) $$\begin{array}{c} |\frac{N(a|x^{n})}{n}-P_{X}\left(a\right)|\leq \delta P_{X}\left(a\right)\;\mathrm{for}\;\mathrm{all}\;a\in X \end{array}$$

where $N(a|x^{n})$ is the number of occurrences of symbol *a* in the sequence $x^{n}$.

A Bernoulli distributed random variable with parameter α∈[0,1] is denoted as Bern(α). Similarly, a binary symmetric channel (BSC) with crossover probability γ∈[0,1] is denoted as BSC(γ). We define the ∗-operator as p∗q=(1−2q)p+q for any p,q∈[0,1]. Moreover, we define the binary entropy function $H_{b}\left(q\right)=-q\log q-\left(1-q\right)\log\left(1-q\right)$.

## 2. Problem Definitions

**Figure 1:**
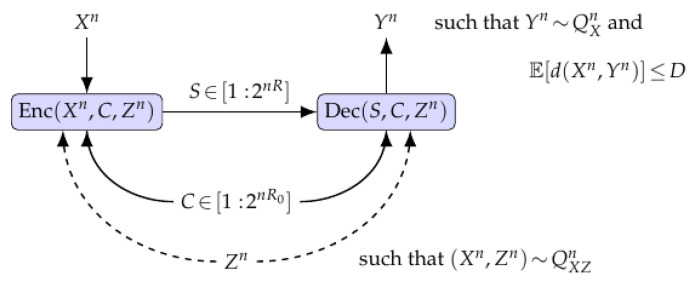
Illustration of a rate–distortion–perception (RDP) model used for neural image compression. The decoder output $Y^{n}$ is required to follow a distribution close to the source probability law $Q_{X}^{n}$, which enforces a realism constraint that reflects perceptual quality. At the same time, the expected distortion between $X^{n}$ and $Y^{n}$ must remain small to reduce the image quality loss due to compression, and the communication rate *R* should be minimized for any fixed common randomness rate $R_{0}\geq 0$ in the finite-blocklength regime to capture low-latency systems. The model also includes an extension in which side information (SI) $Z^{n}$, with $\left(X^{n},Z^{n}\right)∼Q_{XZ}^{n}$, is available at both the encoder and the decoder.

We consider a point-to-point RDFC setup, e.g., for neural image compression, whose goal is to produce perceptually realistic reconstructions subject to communication-rate and distortion constraints; see Figure 1. The encoder observes an input image sequence $X^{n}\in X^{n}$, where X is a finite alphabet, and has access to common randomness $C\in [1:2^{nR_{0}}]$ that is shared with the decoder and is uniformly distributed and independent of $X^{n}$. Such common randomness may be generated, for example, using physical unclonable functions [33]. The encoder applies a mapping $S=\mathrm{Enc}\left(X^{n},C\right)\in \left[1:2^{nR}\right]$. Throughout, we assume that the index *S* is delivered reliably (e.g., using suitable channel coding techniques), and we therefore characterize the compression resources $\left(R,R_{0}\right)$ required to meet distortion and perception constraints at blocklength *n*. A joint RDP-channel analysis of RDP over noisy channels is outside the scope of this paper and would require additional fundamental results, which can be provided by extending the asymptotic results in [34].

Then, given (S,C), the decoder outputs a reconstruction image $Y^{n}=\mathrm{Dec}\left(S,C\right)\in X^{n}$ satisfying the following:

(i) The induced distribution $P_{Y^{n}}$, where we have $y^{n}∼P_{Y^{n}}$, approximates the source distribution $Q_{X}^{n}$;
(ii) The communication rate *R* is as small as possible for a given common randomness rate $R_{0}$; and
(iii) The distortion between $X^{n}$ and $Y^{n}$ is minimized.

We also study an extension in which both terminals have access to correlated side information (SI) $Z^{n}\in Z^{n}$, which is jointly distributed with $X^{n}$ according to $\left(X^{n},Z^{n}\right)∼Q_{XZ}^{n}$.

By fixing a blocklength n≥1, we obtain a finite-blocklength characterization of the RDP region, which considers low-latency deep learning-based image compression. In this setting, we define three non-asymptotic RDP regions: a rate region without a secrecy constraint or SI, a region in which SI is available at both terminals, and a secure RDP region in which an eavesdropper may observe the publicly transmitted index *S*. Throughout, $\epsilon_{r},\epsilon_{D},\epsilon_{\sec}>0$ denote fixed parameters for realism, distortion, and secrecy, respectively.

**Definition 1.** 
*An RDP tuple $\left(R,R_{0},D\right)$ is said to be ($\epsilon_{r},\epsilon_{D},n$)-achievable for $Q_{X}$ if there exist an encoder and a decoder satisfying*

(5) $$\begin{array}{cccc} \; & {∥P_{Y^{n}}-Q_{X}^{n}∥}_{\mathrm{TV}}\leq \epsilon_{r} & \; & \left(\mathrm{realism}\right) \end{array}$$

(6) $$\begin{array}{cccc} \; & E\left[d(X^{n},Y^{n})\right]\;\leq \;D\;+\;\epsilon_{D} & \; & \left(\mathrm{distortion}\right) \end{array}$$

*where $d:X\times X\to [0,d_{\max}]$ is any per-letter distortion metric and we define*

(7) $$\begin{array}{c} d\left(x^{n},y^{n}\right)≜\frac{1}{n}\sum_{i=1}^{n}d\left(x_{i},y_{i}\right). \end{array}$$

*The closure of the set of all ($\epsilon_{r},\epsilon_{D},n$)-achievable $\left(R,R_{0},D\right)$ tuples is defined as the non-asymptotic RDP region $R_{\mathrm{RDP}}$.*

We next introduce the non-asymptotic RDP with SI region $R_{\mathrm{RDPSI}}$, defined for the case in which both the encoder and decoder have access to SI $Z^{n}$. As mentioned above, the SI models additional, correlated observations of the source (for example, previously acquired frames), which can be exploited to substantially improve the RDP performance, as discussed below.

**Definition 2.** 
*Given shared SI $Z^{n}$, an RDP tuple $\left(R,R_{0},D\right)$ is said to be ($\epsilon_{r},\epsilon_{D},n$)-achievable for $Q_{XZ}$ if there exist an encoder and a decoder satisfying (5) and (6).*

*The closure of the set of all ($\epsilon_{r},\epsilon_{D},n$)-achievable $\left(R,R_{0},D\right)$ tuples is defined as the non-asymptotic RDP with SI region $R_{\mathrm{RDPSI}}$.*

We now introduce a secrecy constraint that bounds the information an eavesdropper observing the index *S* can infer about the reconstruction image $Y^{n}$. This requirement is particularly relevant in applications such as generative artificial intelligence for artistic digital content, where the reconstructed image $Y^{n}$ itself should be protected.

**Definition 3.** 
*An RDP tuple $\left(R,R_{0},D\right)$ is said to be ($\epsilon_{r},\epsilon_{D},\epsilon_{\sec},n$)-achievable for $Q_{X}$ under a strong secrecy constraint if there exist an encoder and a decoder satisfying (5), (6), and*

(8) $$\begin{array}{cccc} \; & \left|\right|P_{SY^{n}}-P_{S}P_{Y^{n}}{\left|\right|}_{\mathrm{TV}}\leq \epsilon_{\sec} & \; & (\mathrm{strong}\;\mathrm{secrecy}). \end{array}$$

*The closure of the set of all ($\epsilon_{r},\epsilon_{D},\epsilon_{\sec},n$)-achievable $\left(R,R_{0},D\right)$ tuples is defined as the non-asymptotic secure RDP region $R_{\mathrm{SRDP}}$.*

The requirement in (8) corresponds to a strong secrecy constraint, as it limits absolute information leakage (known in the cryptographic literature as “noisy leakage” [35,36]) rather than leakage normalized by *n*, which forms the basis of the classical weak secrecy notion used, for example, in [37].

We now present finite-blocklength achievable RDP regions that serve as inner bounds for $R_{\mathrm{RDP}}$, $R_{\mathrm{RDPSI}}$, and $R_{\mathrm{SRDP}}$. Furthermore, we characterize the asymptotic secure RDP region under a perfect realism constraint that is stricter than the near-perfect realism constraint in (5).

## 3. Main Results

Before presenting the main results, we note that the theorems in this section serve complementary purposes. The first theorem characterizes the fundamental finite-blocklength RDP limits without additional resources, the second one incorporates SI to capture, e.g., contextual knowledge available at both terminals, and the third one introduces a secrecy constraint to model adversarial settings. Moreover, the fourth theorem identifies the corresponding asymptotic RDP region under a perfect realism constraint. A detailed comparison of these regions and their implications is given in Section 4.

Following the notation in [38,39,40], we define the channel dispersions associated with the test channels $P_{U|X}$ and $P_{U|Y}$ as

(9) $$\begin{array}{cc} V_{U|X} & =E_{P_{UX}}\left[\mathrm{Var}[ı(U,X)|U]\right], \end{array}$$

(10) $$\begin{array}{cc} V_{U|Y} & =E_{P_{UY}}\left[\mathrm{Var}[ı(U,Y)|U]\right]. \end{array}$$

Here, the test channels $P_{U|X}$ and $P_{U|Y}$ relate the variables *X* and *Y* in Figure 1 to an auxiliary random variable *U* used in representing the code construction. Define

(11) $$\begin{array}{c} \mu_{xy}=\min_{\left(x,y\right)\in \mathrm{supp}\left(P_{XY}\right)}P_{XY}\left(x,y\right). \end{array}$$

We now characterize a non-asymptotic RDP region that is ($\epsilon_{r},\epsilon_{D},n$)-achievable, which establishes an achievable RDP trade-off at finite blocklengths.

**Theorem 1.** 
*An ($\epsilon_{r},\epsilon_{D},n$)-achievable non-asymptotic RDP region is the union of the rate tuples $\left(R,R_{0},D\right)$ over all distributions $P_{XUY}=Q_{X}P_{UY|X}$, such that we have the rate constraints*

(12) $$\begin{array}{cc} \; & R\geq {\left[I\left(U;X\right)+Q^{-1}\left(\epsilon_{r}\;+\;O(\frac{1}{\sqrt{n}})\right)\sqrt{\frac{V_{U|X}}{n}}+O\left(\frac{\log n}{n}\right)\right]}^{+}, \end{array}$$

(13) $$\begin{array}{cc} \; & R+R_{0}\geq {\left[I\left(U;Y\right)+Q^{-1}\left(\epsilon_{r}\;+\;O(\frac{1}{\sqrt{n}})\right)\sqrt{\frac{V_{U|Y}}{n}}+O\left(\frac{\log n}{n}\right)\right]}^{+} \end{array}$$

*where X−U−Y form a Markov chain, and the distortion constraint*

(14) $$\begin{array}{cc} \; & D\geq E\left[d\left(X,Y\right)\right]-\delta_{D}-\epsilon^{\prime }\left(\epsilon_{r}\right)d_{\max} \end{array}$$

*where we have some function $\epsilon^{\prime }\left(\epsilon_{r}\right)>0$ such that $\epsilon^{\prime }\left(\epsilon_{r}\right)\to 0$ if $\epsilon_{r}\to 0$ and*

(15) $$\begin{array}{c} \epsilon_{D}=\delta_{D}\left(1+D+\delta_{D}\right)+2{\left|X\right|}^{2}e^{-2n\delta_{D}^{2}\mu_{xy}^{2}}d_{\max}. \end{array}$$

*It suffices to consider $\left|U\right|\leq {\left|X\right|}^{2}+1$.*

**Proof.**  The achievability proof relies on non-asymptotic random binning techniques developed in [39,41,42], which develop finite-blocklength code constructions as in [38,43,44] for the output statistics of random binning (OSRB) method [45,46]. In what follows, we describe the adaptations and refinements specific to our setting and indicate the places where the arguments depart from the standard approaches. For comprehensive treatments of the standard proof steps, we refer the reader to [25,39,42,47], which we do not reproduce here for convenience.

Fix a distribution $P_{XUY}=Q_{X}P_{UY|X}$ that satisfies the distortion constraint

(16) $$\begin{array}{c} E\left[d\left(X,Y\right)\right]\leq D+\delta_{D} \end{array}$$

where $\delta_{D}\geq 0$ satisfies (15). The difference between the expected distortion $E[d\left(X^{n},Y^{n}\right)]$ under $P_{XY}^{n}$ and under the synthesized joint probability distribution can be bounded by $\epsilon^{\prime }\left(\epsilon_{r}\right)d_{\max}$, where $\epsilon^{\prime }\left(\epsilon_{r}\right)>0$ is independent of *n* and $\epsilon^{\prime }\left(\epsilon_{r}\right)\to 0$ if $\epsilon_{r}\to 0$. This bound follows from similar arguments to ([47], Section IV-G and Proposition 53). Note that ([47], Proposition 53) introduces two additional error terms, beyond the realism parameter $\epsilon_{r}$, which must also vanish in order to guarantee that $\epsilon^{\prime }\left(\epsilon_{r}\right)\to 0$. While this vanishing behavior constitutes a necessary condition, any asymptotically optimal code design will, by construction, satisfy it. For this reason, and in the interest of clarity, we provide only a sufficient condition for ensuring $\epsilon^{\prime }\left(\epsilon_{r}\right)\to 0$, without explicitly specifying the exact dependence on all parameters; see ([47], Proposition 53) for the exact error terms. Thus, in the following analysis, we consider i.i.d. sequences by accounting for this difference.

Define the error event that the sequences $\left(X^{n},Y^{n}\right)$ are not $\delta_{D}$-letter typical as

(17) $$\begin{array}{c} E=\{\left(X^{n},Y^{n}\right)\notin T_{\delta_{D}}^{n}\left(P_{XY}\right)\}. \end{array}$$

Using similar steps as in [48], we obtain (15), given (6), since we have

(18) $$\begin{array}{cc} \; & E\left[d\left(X^{n},Y^{n}\right)\right]=\mathrm{Pr}\left[E^{c}\right]\;E\left[d\left(X^{n},Y^{n}\right)|E^{c}\right]+\mathrm{Pr}\left[E\right]\;E\left[d\left(X^{n},Y^{n}\right)|E\right]\\ \; & \overset{\left(a\right)}{\leq }\mathrm{Pr}\left[E^{c}\right]\;\left(1+\delta_{D}\right)\;E\left[d\left(X,Y\right)\right]+\mathrm{Pr}\left[E\right]\;d_{\max}\\ \; & \overset{\left(b\right)}{\leq }\left(1+\delta_{D}\right)\;\left(D+\delta_{D}\right)+2{\left|X\right|}^{2}e^{-2n\delta_{D}^{2}\mu_{xy}^{2}}\;d_{\max} \end{array}$$

where (a) follows from the typical average lemma ([49], p. 26); since the distortion metric is per-letter with bound $d_{\max}$, (b) follows (16). A bound on Pr[E] given in ([50], Equation (6.34)), which can be applied as a per-letter estimator, is used.

We next prove that there exist non-asymptotic random binning schemes simultaneously satisfying the realism and distortion constraints. Generate an auxiliary random variable sequence $U^{n}$ in an i.i.d. manner such that we have a joint probability distribution $P_{XU}=Q_{X}P_{U|X}$. Following the structure of the OSRB method, we first analyze a source coding problem (Protocol A). In Protocol A, the encoder maps $U^{n}$ independently and uniformly to three random bin indices

(19) $$\begin{array}{c} S\in \left[1\;:\;2^{nR}\right],\;F\in \left[1\;:\;2^{n\tilde{R}}\right],\;C\in \left[1\;:\;2^{nR_{0}}\right]. \end{array}$$

In this protocol, the index *F* represents the public choice of encoder–decoder pairs. Using a mismatch stochastic likelihood coder as the decoder, as in ([42], p. 3) and ([39], Equation (12)), that observes (S,F,C), we can bound the expected error probability averaged over the random binning ensemble.

Now, the rate constraints are imposed to ensure that the encoder–decoder pair aims to satisfy the following, for suitable nonnegative error parameters $\epsilon_{\mathrm{ind}}\left(n\right)$, $\epsilon_{\mathrm{ind}}^{\prime }\left(n\right)$, and $\epsilon_{\mathrm{dec}}\left(n\right)$ that vanish as n→∞, with the penalties for finite blocklengths:

(i) (C,F) are almost independent of $X^{n}$ such that we have
  (20) $$\begin{array}{c} {∥P_{X^{n}CF}-Q_{X}^{n}P_{CF}∥}_{\mathrm{TV}}\leq \epsilon_{\mathrm{ind}}\left(n\right); \end{array}$$
(ii) (C,F,S) almost recover $U^{n}$ such that we have
  (21) $$\begin{array}{c} \mathrm{Pr}\left[{\hat{U}}^{n}\left(C,F,S\right)\neq U^{n}\right]\leq \epsilon_{\mathrm{dec}}\left(n\right); \end{array}$$
(iii) *F* is almost independent of $Y^{n}$ such that we have
  (22) $$\begin{array}{c} {∥P_{Y^{n}F}-Q_{X}^{n}P_{F}∥}_{\mathrm{TV}}\leq \epsilon_{\mathrm{ind}}^{\prime }\left(n\right). \end{array}$$

To impose constraints that ensure near independence, we apply ([42], Theorem 1). Similarly, to impose reliable sequence reconstruction constraints, we apply ([42], Theorem 2). These steps yield rate constraints on $\left(\tilde{R},R,R_{0}\right)$ given in Theorem 1, derived by applying Berry–Esseen Theorem such that the total variation distances between the target and observed probability distributions are bounded by a fixed value. This analysis corresponds to Protocol B, a channel coding problem dual to our problem with extra randomness *F* in Protocol A. Furthermore, the proof of the realism constraint (5) follows by applying the soft covering lemma ([13], Lemma IV.1) as in the achievability proof of ([25], Theorem 2).

To eliminate extra randomness *F* such that $\tilde{R}$ is also eliminated from the rate constraints, we show that a fixed realization F=f can be agreed upon publicly by the encoder and decoder, following by applying arguments similar to those in [39,45]. Finally, by selecting the free parameters similar to the choices in ([39], Equation (36)), we obtain (12) and (13). This follows because in the above analysis, the quantities $\epsilon_{\mathrm{ind}}\left(n\right)$, $\epsilon_{\mathrm{ind}}^{\prime }\left(n\right)$, and $\epsilon_{\mathrm{dec}}\left(n\right)$ are auxiliary design parameters that control, respectively, the independence conditions in (20) and (22) and the decoding error in (21). For any fixed blocklength *n* and target realism parameter $\epsilon_{r}$, we choose these parameters in such a way that $2\left(\epsilon_{\mathrm{ind}}\left(n\right)+\epsilon_{\mathrm{ind}}^{\prime }\left(n\right)+5\epsilon_{\mathrm{dec}}\left(n\right)\right)\leq \left(\epsilon_{r}+O\;\left(\frac{1}{\sqrt{n}}\right)\right)$, as in ([39], Equations (23b) and (36)). The finite-length OSRB theorems in ([42], Theorems 1 and 2) then guarantee that, whenever the rate pair $\left(R,R_{0}\right)$ satisfies the bounds in (12) and (13), there exists a code for which (20)–(22) hold with these parameter choices.

The cardinality bound on the auxiliary random variable *U* follows from the support lemma ([5], Lemma 15.4). Since we have Y=X, preserving the joint probability distribution $P_{XY}$ requires $\left(|X{|}^{2}-1\right)$ continuous real-valued constraints. Moreover, the two expressions corresponding to the lower bounds in (12) and (13) must be preserved. Consequently, the support lemma implies that it is sufficient to restrict $\left|U\right|\leq {\left|X\right|}^{2}+1$. □

Note that the asymptotic counterparts of the bounds in (12)–(15) recover the RDP region in ([26], Theorem 6) that extends ([51], Theorems 1 and 5).

**Remark 1.** 
*Taking the limit n→∞ in Theorem 1 recovers the asymptotic RDP region. In this regime, the rate R=I(U;X) is achievable whenever the decoder has access to enough common randomness, specifically when $R_{0}\geq I\left(U;Y\right)-I\left(U;X\right)$. Because H(X) is the minimum rate required for any lossless compression method, the ratio H(X)/I(U;X) captures the potential rate reductions attainable through RDP-based schemes. As shown in [6], exploiting common randomness in the RDFC framework can yield rate reductions exceeding a factor of 214 in differential privacy applications. Similar improvements can arise in the RDP setting, depending on the source distribution $Q_{X}$ and the distortion measure d(·,·). Moreover, we note that communication rate comparisons with lossless compression methods must be interpreted relative to distortion level (and perception constraint). While lossless coding enforces asymptotically negligible distortion, RDP schemes operate at nonzero minimal distortion levels characterized by an RDP distortion-rate function that depends on the communication and common-randomness rates.*

Denote channel dispersions for test channels $P_{U|XZ}$ and $P_{U|YZ}$ as

(23) $$\begin{array}{cc} V_{U|XZ} & =E_{P_{UXZ}}\left[\mathrm{Var}[ı(U,XZ)|U]\right], \end{array}$$

(24) $$\begin{array}{cc} V_{U|YZ} & =E_{P_{UYZ}}\left[\mathrm{Var}[ı(U,YZ)|U]\right]. \end{array}$$

Define

(25) $$\begin{array}{c} \mu_{xy|z}=\min_{\left(x,y,z\right)\in \mathrm{supp}\left(P_{XY|Z}\right)}P_{XY|Z}\left(x,y|z\right). \end{array}$$

Now, we establish an ($\epsilon_{r},\epsilon_{D},n$)-achievable non-asymptotic RDP with SI region, which extends the non-asymptotic RDP region by quantifying how shared SI alters the achievable trade-offs.

**Theorem 2.** 
*An ($\epsilon_{r},\epsilon_{D},n$)-achievable non-asymptotic RDP with SI region is the union of the rate tuples $\left(R,R_{0},D\right)$ over all distributions $P_{XZUY}=Q_{XZ}P_{UY|XZ}$, such that we have the distortion constraint (14) with*

(26) $$\begin{array}{c} \epsilon_{D}=\delta_{D}\left(1+D+\delta_{D}\right)+{2|X|}^{2}\left|Z\right|e^{-2n\delta_{D}^{2}\mu_{xy|z}^{2}}d_{\max} \end{array}$$

*and the rate constraints*

(27) $$\begin{array}{cc} \; & R\geq {\left[I\left(U;X|Z\right)+Q^{-1}\left(\epsilon_{r}\;+\;O(\frac{1}{\sqrt{n}})\right)\sqrt{\frac{V_{U|XZ}}{n}}+O\left(\frac{\log n}{n}\right)\right]}^{+}, \end{array}$$

(28) $$\begin{array}{cc} \; & R+R_{0}\geq {\left[I\left(U;Y|Z\right)-H\left(Z|Y\right)+\;Q^{-1}\left(\epsilon_{r}\;+\;O(\frac{1}{\sqrt{n}})\right)\sqrt{\frac{V_{U|YZ}}{n}}+O\left(\frac{\log n}{n}\right)\right]}^{+} \end{array}$$

*where X−(U,Z)−Y form a Markov chain. It suffices to consider $\left|U\right|\leq {\left|X\right|}^{2}\left|Z\right|+1$.*

**Proof.**  The achievability proof follows by using a coding method similar to the code construction designed in the achievability proof for Theorem 1, where, this time, we use a different codebook for each realization $z^{n}\in Z^{n}$. Using a different codebook per realization is similarly applied to an asymptotic RDP with SI case in ([47], Section IV-B), which leverages a strong coordination with SI result in ([13], Corollary VII.5).

We then impose rate constraints to asymptotically satisfy the following: (i) (C,F) are almost independent of $\left(X^{n},Z^{n}\right)$; (ii) $\left(C,F,S,Z^{n}\right)$ almost recover $U^{n}$; and (iii) using the soft-covering lemma with SI ([13], Corollary VII.5) and recognizing that the term (I(U;Y|Z)−H(Z|Y)) in (28) is equal to (I(U,Z;Y)−H(Z)) where the latter is the form used in ([13], Corollary VII.5), we satisfy the realism constraint (5); see also ([47], pp. 10–11). Moreover, the elimination of the extra randomness *F* and the proof of the cardinality bound follow similarly to the steps given in the achievability proof for Theorem 1. □

We remark that by allowing n→∞, the bounds in (14) and (26)–(28) recover the asymptotic RDP with SI region in ([47], Theorem 8).

We next establish an ($\epsilon_{r},\epsilon_{D},\epsilon_{\sec},n$)-achievable non-asymptotic secure RDP region, which characterizes the stricter constraints imposed on the non-asymptotic RDP region when strong secrecy against an eavesdropper is required.

**Theorem 3.** 
*An ($\epsilon_{r},\epsilon_{D},\epsilon_{\sec},n$)-achievable non-asymptotic secure RDP region is the union of the rate tuples $\left(R,R_{0},D\right)$ over all distributions $P_{XUY}=Q_{X}P_{UY|X}$, such that, for any θ∈[0,1], we have the distortion constraint (14) with (15) and the rate constraints*

(29) $$\begin{array}{cc} \; & R\geq {\left[I\left(U;X\right)+Q^{-1}\left(\theta (\epsilon_{r}\;+\;O(\frac{1}{\sqrt{n}}))\right)\sqrt{\frac{V_{U|X}}{n}}+O\left(\frac{\log n}{n}\right)\right]}^{+}, \end{array}$$

(30) $$\begin{array}{cc} \; & R_{0}\geq {\left[I\left(U;Y\right)+\;Q^{-1}\left(\left(1\;-\;\theta \right)(\epsilon_{\sec}\;+\;O(\frac{1}{\sqrt{n}}))\right)\sqrt{\frac{V_{U|Y}}{n}}+O\left(\frac{\log n}{n}\right)\right]}^{+} \end{array}$$

*where X−U−Y form a Markov chain. It suffices to consider $\left|U\right|\leq {\left|X\right|}^{2}+1$.*

**Proof.**  The structure of the achievability proof steps parallels that of Theorem 1, but the secure setting requires demonstrating that a non-asymptotic random binning scheme can be constructed to meet the two constraints

(31) $$\begin{array}{cc} \; & {∥P_{Y^{n}}-Q_{X}^{n}∥}_{\mathrm{TV}}\leq \theta \epsilon_{r}, \end{array}$$

(32) $$\begin{array}{cc} \; & \left|\right|P_{SY^{n}}-P_{S}P_{Y^{n}}{\left|\right|}_{\mathrm{TV}}\leq \left(1-\theta \right)\epsilon_{\sec} \end{array}$$

for any θ∈[0,1]; see also ([42], Theorem 4). To this end, we employ the same random code ensemble used in Theorem 1, but instead of enforcing almost-independence between *F* and $Y^{n}$, we require that the pair (S,F) be almost independent of $Y^{n}$. Proceeding through the OSRB-based analysis with this modified independence condition yields the bounds in (29) and (30). Finally, the cardinality bound on the auxiliary random variable follows from the same arguments used in the proof of Theorem 1, since $P_{XY}$ and the expressions appearing in (29) and (30) must be preserved. □

Taking the limit n→∞, the asymptotic forms of the bounds in (14), (15), (29), and (30) coincide with the asymptotic secure RDP region derived in [34]. This recovery follows once the factors θ and (1−θ) in (29) and (30) are removed.

The condition in (5) is commonly described as a *near-perfect* (or *strong*) realism constraint, since the parameter $\epsilon_{r}$ can be driven arbitrarily close to zero as n→∞, although it never vanishes exactly. In contrast, a *perfect realism* constraint imposes

(33) $$\begin{array}{c} {∥P_{Y^{n}}-Q_{X}^{n}∥}_{\mathrm{TV}}=0\;\;(\mathrm{perfect}\;\mathrm{realism}) \end{array}$$

and under this stricter requirement the asymptotic secure RDP region admits the following characterization.

**Theorem 4.** 
*The asymptotic secure RDP region with perfect realism constraint is the union of the rate tuples $\left(R,R_{0},D\right)$ over all distributions $P_{XUY}=Q_{X}P_{UY|X}$, such that we have*

(34) $$\begin{array}{cc} \; & R\geq I(U;X), \end{array}$$

(35) $$\begin{array}{cc} \; & R_{0}\geq I\left(U;Y\right), \end{array}$$

(36) $$\begin{array}{cc} \; & D\geq E[d(X,Y)] \end{array}$$

*where X−U−Y form a Markov chain. It suffices to consider $\left|U\right|\leq {\left|X\right|}^{2}+1$.*

**Proof.**  The asymptotic secure RDP region under near-perfect realism is obtained by taking n→∞ in the bounds (14), (15), (29), and (30), which recovers the characterization given in [34]. This asymptotic result is then combined with ([25], Theorem 1), which shows that a rate triple $\left(R,R_{0},D\right)$ is achievable under near-perfect realism if and only if it is achievable under perfect realism. Applying this equivalence to the region of [34] yields Theorem 4. □

**Remark 2.** 
*The optimal code constructions for the asymptotic secure RDP regions under perfect and near-perfect realism are not necessarily the same even if they impose the same bounds.*

## 4. Comparisons and Discussions

From a system-design perspective, the derived RDP regions clarify how communication rate, common randomness rate, SI, and distortion act as controllable resources in semantic compression systems. The blocklength-rate product nR determines the communication load in low-latency regimes, shared SI reduces the required communication and common randomness resources, and the common randomness rate $R_{0}$ can reflect the availability of shared generative seeds or synchronized models. Moreover, the finite-blocklength and secrecy-constrained regions quantify the additional resource demands imposed by latency and security requirements, respectively. The achievable regions derived in Section 3 can then be used operationally by fixing one design parameter and inspecting the induced trade-off between the remaining parameters, which is the workflow also used in this section to analyse the effects of each parameter across the baseline RDP, RDP with SI, and secure RDP scenarios.

The availability of SI $Z^{n}$ at both the encoder and decoder enlarges the achievable RDP region through two complementary mechanisms. First, SI can lower the required communication rate *R*; compare (12) and (27). A similar effect appears in the leading term of the sum-rate constraint in (28). Second, the component of $Z^{n}$ that cannot be inferred from $Y^{n}$ effectively behaves as additional common randomness, which can reduce the required common-randomness rate; compare (13) and (28). This dual role of SI, also emphasized in [47], is precisely what Theorems 1 and 2 quantify in our non-asymptotic results. For instance, if $Z^{n}$ is independent of $\left(X^{n},C\right)$, SI no longer decreases the communication rate but acts as an independent source of common randomness with rate H(Z). In this regime, the achievable RDP with SI region in Theorem 2 reduces to the region in Theorem 1, with the distinction that the required common-randomness rate can be decreased by H(Z); see additionally ([47], p. 10).

Compared with the non-secure RDP region of Theorem 1, the secure RDP region in Theorem 3 differs both in structure and in the resulting bounds. Without secrecy constraint, the description involves a single sum-rate constraint, which yields a larger achievable region. With secrecy constraint, in contrast, the rates are bounded separately to meet realism and secrecy simultaneously. For example, suppose

(37) $$\begin{array}{cc} \; & X∼\mathrm{Bern}\left(0.5\right),\;\;Z\;=\;Ø,\;\;P_{X|U}∼\mathrm{BSC}\left(\gamma \right),\;\;P_{Y|U}∼\mathrm{BSC}\left(\eta \right) \end{array}$$

and a uniformly distributed binary auxiliary random variable *U*. For this example scenario, we compare achievable tuples for the asymptotic non-secure and secure RDP regions, for which we obtain

(38) $$\begin{array}{cc} \; & I\left(U;X\right)=1-H_{b}\left(\gamma \right), \end{array}$$

(39) $$\begin{array}{cc} \; & I\left(U;Y\right)=1-H_{b}\left(\eta \right), \end{array}$$

(40) $$\begin{array}{cc} \; & D\geq \gamma *\eta . \end{array}$$

**Figure 2:**
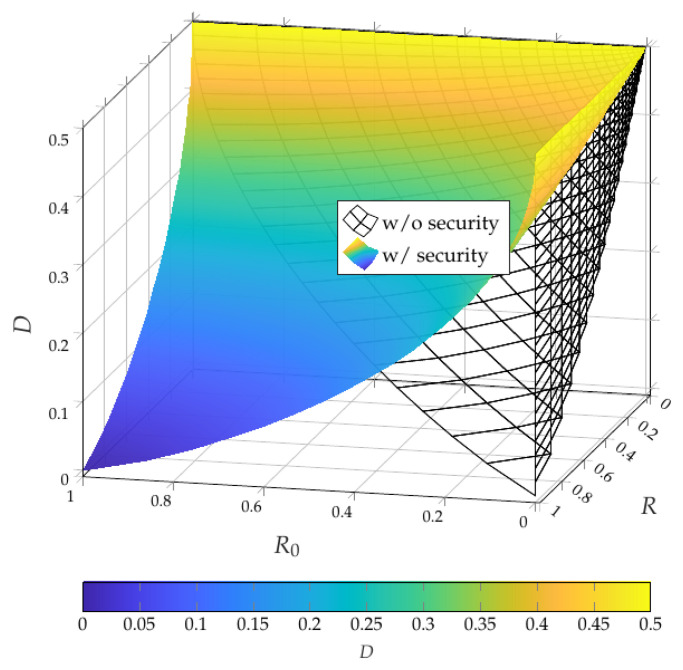
Asymptotic RDP Pareto boundaries for the binary example in Section 4. The colored surface shows the Pareto boundary of the achievable secure RDP region, while the wireframe depicts the corresponding Pareto boundary of the achievable non-secure RDP region.

Taking the union over all γ,η∈[0,1] for the corresponding rate bounds results in the achievable non-secure and secure RDP regions, whose Pareto boundaries are depicted in Figure 2. This figure highlights the significant shrinkage of the achievable rate region when secrecy is imposed, requiring larger communication *R* and common-randomness $R_{0}$ rates. Moreover, unlike the non-secure case where $R_{0}=0$ is feasible, secure RDP requires a strictly positive common randomness rate for non-trivial distortion levels. Thus, this comparison highlights the fundamental performance loss intrinsic to strong secrecy in, e.g., RDP-based neural compression systems. Note that these evaluation steps can be extended for continuous-alphabet random variables, such as Gaussian vector sources under standard distortion and perception measures, as considered in [52,53] for closely related problems.

Furthermore, the secrecy requirement amplifies the finite-blocklength penalties, since the realism $\epsilon_{r}$ and secrecy $\epsilon_{\sec}$ parameters enter the bounds through the scaling factors θ and (1−θ). As a result, the corresponding additive terms become larger than in the non-secure case. These effects highlight the inherent cost of enforcing strong secrecy, as achieving it typically necessitates increased communication and common-randomness rates, with the overhead being most pronounced at short blocklengths. Such trade-offs are especially important in applications that must jointly account for security, latency, distortion, and realism when choosing between secure and non-secure neural compression methods.

As n→∞, the finite-blocklength penalties disappear and we recover the asymptotic rate regions. For finite *n*, however, the achievable bounds include correction terms of the form $Q^{-1}\left(\epsilon \right)\sqrt{V/n}$, which reflect statistical fluctuations that arise at finite blocklengths. These terms exhibit the same functional dependence as those appearing in finite-blocklength channel and source coding [38,43], although the analysis of RDP problems requires different proof techniques due to the perception constraint. Comparing the finite-*n* RDP regions with their asymptotic counterparts shows that, at finite blocklengths, achieving a given distortion level requires larger rates, and for a fixed-rate tuple the best attainable distortion is higher than in the asymptotic limit. Such insights are essential for understanding the performance of neural image compression systems that must function reliably under stringent constraints on latency, realism, and distortion.

## 5. Conclusions

Within the RDFC framework, this work broadened classical rate–distortion analyses by incorporating perceptual quality requirements and strong secrecy guarantees. We derived finite-blocklength achievable regions for the RDP problem and examined their asymptotic limits, providing a theoretical basis for low-latency, high-fidelity, and secure neural image compression under realism constraints. In addition, we showed that shared side information can substantially enlarge the achievable regions by reducing the required amount of both communication and common-randomness rates. Although the assumption of stationary memoryless sources, considered here, is idealized, it is well-accepted in the machine learning literature on RDP tradeoffs, enabling tractable analysis and insightful benchmarks; see, for instance, [20]. Extensions to more general non-i.i.d. models using information-spectrum methods [54] are possible but typically yield far less tractable expressions, so we restricted attention to the i.i.d. setting for clarity and interpretability.

From an application perspective, the obtained bounds guide the design of ultra-efficient neural image compression schemes that must simultaneously meet requirements on latency, realism, distortion, and security. The strong secrecy guarantees, which provide robustness even against adversaries with quantum capabilities, align the RDP framework with the broader shift toward quantum-safe communications. Future work includes treating noisy transmission channels, which would advance the theory of joint RDP-channel coding and further extend the practical relevance of RDP methods in emerging deep learning-based systems.

## Data Availability

The data presented in this study are available on request from the corresponding author.

## Abbreviations

The following abbreviations are used in this manuscript:

BSC	binary symmetric channel
i.i.d.	independent and identically distributed
OSRB	output statistics of random binning
PLS	physical-layer security
RDFC	randomized distributed function computation
RDP	rate–distortion–perception
SI	side information
TV	total variation

## References

[1] Gündüz D., Chiariotti F., Huang K., Kalør A.E., Kobus S., Popovski P. (2023). Timely and Massive Communication in 6G: Pragmatics, Learning, and Inference. IEEE BITS Inf. Theory Mag..

[2] Gündüz D., Qin Z., Aguerri I.E., Dhillon H.S., Yang Z., Yener A., Wong K.K., Chae C.B. (2022). Beyond Transmitting Bits: Context, Semantics, and Task-Oriented Communications. IEEE J. Sel. Areas Commun. (JSAC).

[3] Dobrushin R., Tsybakov B. (1962). Information transmission with additional noise. IRE Trans. Inf. Theory (T-IT).

[4] Berger T. (2003). Rate-distortion Theory. Wiley Encyclopedia of Telecommunication.

[5] Csiszár I., Körner J. (2011). Information Theory: Coding Theorems for Discrete Memoryless Systems.

[6] Günlü O. Randomized Distributed Function Computation with Semantic Communications: Applications to Privacy. Proceedings of the IEEE International Workshop on Information Forensics and Security (WIFS).

[7] Flamich G., Havasi M., Hernández-Lobato J.M. (2020). Compressing images by encoding their latent representations with relative entropy coding. Adv. Neural Inf. Process. Sys. (NeurIPS).

[8] Havasi M., Peharz R., Hernández-Lobato J.M. Minimal random code learning: Getting bits back from compressed model parameters. Proceedings of the International Conference on Learning Representation (ICLR).

[9] Isik B., Pase F., Gunduz D., Koyejo S., Weissman T., Zorzi M. Adaptive compression in federated learning via side information. Proceedings of the International Conference on Artificial Intelligence and Statistics (AISTATS).

[10] Hegazy M., Leluc R., Li C.T., Dieuleveut A. Compression with exact error distribution for federated learning. Proceedings of the International Conference on Artificial Intelligence and Statistics (AISTATS).

[11] Shah A., Chen W.N., Balle J., Kairouz P., Theis L. Optimal compression of locally differentially private mechanisms. Proceedings of the International Conference on Artificial Intelligence Statistics (AISTATS).

[12] Bergström D., Günlü O. Deep randomized distributed function computation (DeepRDFC): Neural distributed channel simulation. Proceedings of the IEEE International Symposium on Information Theory (ISIT).

[13] Cuff P. (2013). Distributed Channel Synthesis. IEEE Trans. Inf. Theory (T-IT).

[14] Niu X., Bai B., Guo N., Zhang W., Han W. (2025). Rate–Distortion–Perception Trade-Off in Information Theory, Generative Models, and Intelligent Communications. Entropy.

[15] Sayood K. (2017). Introduction to Data Compression.

[16] Günlü O., Skorski M., Poor H.V. Low-latency rate-distortion-perception trade-Off: A randomized distributed function computation application. Proceedings of the 2025 Joint European Conference on Networks and Communications & 6G Summit (EuCNC/6G Summit).

[17] Chen J., Yu L., Wang J., Shi W., Ge Y., Tong W. (2022). On the rate-distortion-perception function. IEEE J. Sel. Areas Inf. Theory (JSAIT).

[18] Matsumoto R. (2018). Introducing the perception-distortion tradeoff into the rate-distortion theory of general information sources. IEICE Commun. Express (ComEX).

[19] Zhang G., Qian J., Chen J., Khisti A. (2021). Universal rate-distortion-perception representations for lossy compression. Adv. Neural Inf. Process. Sys. (NeurIPS).

[20] Blau Y., Michaeli T. Rethinking lossy compression: The rate-distortion-perception tradeoff. Proceedings of the International Conference on Machine Learning (ICML).

[21] Theis L., Wagner A.B. A coding theorem for the rate-distortion-perception function. Proceedings of the Neural Compression: From Information Theory to Applications–Workshop@ ICLR.

[22] Gulrajani I., Ahmed F., Arjovsky M., Dumoulin V., Courville A.C. Improved training of Wasserstein GANs. Proceedings of the Advances in Neural Information Processing Systems 30 (NIPS 2017).

[23] Arjovsky M., Chintala S., Bottou L. Wasserstein generative adversarial networks. Proceedings of the InInternational Conference on Machine Learning (ICML).

[24] Goodfellow I.J., Pouget-Abadie J., Mirza M., Xu B., Warde-Farley D., Ozair S., Courville A., Bengio Y. Generative adversarial nets. Proceedings of the Advances in Neural Information Processing Systems 27 (NIPS 2014).

[25] Wagner A.B. (2022). The rate-distortion-perception tradeoff: The role of common randomness. arXiv.

[26] Hamdi Y., Wagner A.B., Gündüz D. (2024). The Rate-Distortion-Perception Trade-off: The Role of Private Randomness. arXiv.

[27] Bloch M., Günlü O., Yener A., Oggier F., Poor H.V., Sankar L., Schaefer R.F. (2021). An Overview of Information-Theoretic Security and Privacy: Metrics, Limits and Applications. IEEE J. Sel. Areas Inf. Theory (JSAIT).

[28] Günlü O., Bloch M., Schaefer R.F. Secure multi-function computation with private remote sources. Proceedings of the IEEE International Symposium on Information Theory (ISIT).

[29] Post-Quantum Cryptography. https://csrc.nist.gov/projects/post-quantum-cryptography.

[30] Djordjevic I.B. (2022). Physical-Layer Security, Quantum Key Distribution, and Post-Quantum Cryptography. Entropy.

[31] Kalkhoran S.A.A., Letafati M., Erdemir E., Khalaj B.H., Behroozi H., Gündüz D. Secure Deep-JSCC against multiple eavesdroppers. Proceedings of the IEEE Global Communications Conference (GLOBECOM).

[32] Hamdi Y., Wagner A.B., Gündüz D. The Rate-Distortion-Perception Trade-Off with Algorithmic Realism. Proceedings of the IEEE International Symposium on Information Theory (ISIT).

[33] Günlü O., Schaefer R.F., Poor H.V. Biometric and Physical Identifiers with Correlated Noise for Controllable Private Authentication. Proceedings of the IEEE International Symposium on Information Theory.

[34] Åhlgren G., Günlü O. Secure rate-distortion-perception trade-off over channels: A randomized distributed function computation (RDFC) application. Proceedings of the IEEE International Symposium on Information Theory (ISIT).

[35] Dziembowski S., Faust S., Skorski M., Oswald E., Fischlin M. (2015). Noisy Leakage Revisited. Advances in Cryptology-EUROCRYPT 2015.

[36] Brian G., Faonio A., Obremski M., Ribeiro J., Simkin M., Skórski M., Venturi D., Canteaut A., Standaert F.X. (2021). The Mother of All Leakages: How to Simulate Noisy Leakages via Bounded Leakage (Almost) for Free. Advances in Cryptology–EUROCRYPT 2021.

[37] Wyner A.D. (1975). The Wire-tap Channel. Bell Labs Tech. J..

[38] Polyanskiy Y., Poor H.V., Verdú S. (2010). Channel Coding Rate in the Finite Blocklength Regime. IEEE Trans. Inf. Theory (T-IT).

[39] Cervia G., Oechtering T.J., Skoglund M. (*ϵ*, *n*) fixed-length strong coordination capacity. Proceedings of the IEEE Information Theory Workshop (ITW).

[40] Günlü O., Bloch M., Schaefer R.F., Yener A. Nonasymptotic performance limits of low-latency secure integrated sensing and communication systems. Proceedings of the IEEE International Conference on Acoustics, Speech and Signal Processing (ICASSP).

[41] Yassaee M.H., Aref M.R., Gohari A. A technique for deriving one-shot achievability results in network information theory. Proceedings of the IEEE International Symposium on Information Theory (ISIT).

[42] Yassaee M.H., Aref M.R., Gohari A. Non-asymptotic output statistics of random binning and its applications. Proceedings of the IEEE International Symposium on Information Theory (ISIT).

[43] Kostina V., Verdú S. (2012). Fixed-Length Lossy Compression in the Finite Blocklength Regime. IEEE Trans. Inf. Theory.

[44] Tan V.Y.F. Achievable second-order coding rates for the wiretap channel. Proceedings of the IEEE International Conference on Communication Systems (ICCS).

[45] Yassaee M.H., Aref M.R., Gohari A. (2014). Achievability Proof via Output Statistics of Random Binning. IEEE Trans. Inf. Theory (T-IT).

[46] Renes J.M., Renner R. (2011). Noisy Channel Coding via Privacy Amplification and Information Reconciliation. IEEE Trans. Inf. Theory.

[47] Hamdi Y., Wagner A.B., Gündüz D. (2025). Rate-Distortion-Perception Trade-off with Strong Realism Constraints: Role of Side Information and Common Randomness. arXiv.

[48] Welling T., Günlü O., Yener A. Low-latency secure integrated sensing and communication with transmitter actions. Proceedings of the IEEE International Workshop on Signal Processin Advances in Wireless Communications (SPAWC).

[49] Gamal A.E., Kim Y.H. (2011). Network Information Theory.

[50] Kramer G. (2018). Multi-User Information Theory.

[51] Saldi N., Linder T., Yüksel S. (2015). Output Constrained Lossy Source Coding with Limited Common Randomness. IEEE Trans. Inf. Theory (T-IT).

[52] Qian J., Salehkalaibar S., Chen J., Khisti A., Yu W., Shi W., Ge Y., Tong W. (2025). Rate-Distortion-Perception Tradeoff for Gaussian Vector Sources. IEEE J. Sel. Areas Inf. Theory (JSAIT).

[53] Salehkalaibar S., Phan B., Khisti A., Yu W. Rate-Distortion-Perception Tradeoff Based on the Conditional Perception Measure. Proceedings of the Biennial Symposium on Communications (BSC).

[54] Verdú S., Han T.S. (1994). A general formula for channel capacity. IEEE Trans. Inf. Theory (T-IT).

